# Mutation status coupled with RNA-sequencing data can efficiently identify important non-significantly mutated genes serving as diagnostic biomarkers of endometrial cancer

**DOI:** 10.1186/s12859-017-1891-6

**Published:** 2017-12-28

**Authors:** Keqin Liu, Li He, Zhichao Liu, Junmei Xu, Yuan Liu, Qifan Kuang, Zhining Wen, Menglong Li

**Affiliations:** 1 College of Chemistry, Sichuan University, Chengdu, Sichuan China; 2 Biogas Appliance Quality Supervision and Inspection Center, Biogas Institute of Ministry of Agriculture, Chengdu, Sichuan China; 3 Division of Bioinformatics and Biostatistics, National Center for Toxicological Research (NCTR), US Food and Drug Administration (FDA), 3900 NCTR Road, Jefferson, AR 72079 USA

**Keywords:** Endometrial cancer, Somatic mutation, RNA sequencing, Differentially expressed genes, Clinical phenotype characteristics

## Abstract

**Background:**

Endometrial cancers (ECs) are one of the most common types of malignant tumor in females. Substantial efforts had been made to identify significantly mutated genes (SMGs) in ECs and use them as biomarkers for the classification of histological subtypes and the prediction of clinical outcomes. However, the impact of non-significantly mutated genes (non-SMGs), which may also play important roles in the prognosis of EC patients, has not been extensively studied. Therefore, it is essential for the discovery of biomarkers in ECs to further investigate the non-SMGs that were highly associated with clinical outcomes.

**Results:**

For the 9681 non-SMGs reported by the mutation annotation pipeline, there were 1053, 1273 and 395 non-SMGs differentially expressed between the patient groups divided by the clinical endpoints of histological grade, histological type as well as the International Federation of Gynecology and Obstetrics (FIGO) stage of ECs, respectively. In the gene set enrichment analysis, the cancer-related pathways, namely neuroactive ligand-receptor interaction signaling pathway, cAMP signaling pathway and calcium signaling pathway, were significantly enriched with the differentially expressed non-SMGs for all the three endpoints. We further identified 23, 19 and 24 non-SMGs, which were highly associated with histological grade, histological type and FIGO stage, respectively, from the differentially expressed non-SMGs by using the variable combination population analysis (VCPA) approach and found that 69.6% (16/23), 78.9% (15/19) and 66.7% (16/24) of the identified non-SMGs had been previously reported to be correlated with cancers. In addition, the averaged areas under the receiver operating characteristic curve (AUCs) achieved by the predictive models with identified non-SMGs as predictors in predicting histological type, histological grade, and FIGO stage were 0.993, 0.961 and 0.832, respectively, which were superior to those achieved by the models with SMGs as features (averaged AUCs = 0.928, 0.864 and 0.535, resp.).

**Conclusions:**

Besides the SMGs, the non-SMGs reported in the mutation annotation analysis may also involve the crucial genes that were highly associated with clinical outcomes. Combining the mutation status with the gene expression profiles can efficiently identify the cancer-related non-SMGs as predictors for cancer prognostic prediction and provide more supplemental candidates for the discovery of biomarkers.

**Electronic supplementary material:**

The online version of this article (10.1186/s12859-017-1891-6) contains supplementary material, which is available to authorized users.

## Background

Endometrial cancers (ECs) are the most common malignancies among women in the Western world. The prevalence of ECs is increasing [1], with an estimated 60,050 new cases and 10,470 deaths in 2016 [2], likely due to the obesity that is a major risk factor of ECs [3]. ECs can be divided into different subtypes, each exhibiting a unique pathology and different biological behaviour [4].

Somatic mutation is a major factor in tumorigenesis. Recent advances have revealed that mutations in cancer genes are implicated in tumour development and have promoted our understanding of cancer pathology [5]. The standard method employed thus far, is to identify mutated genes based on the frequency of gene mutations in one type of cancer [6]. Mutation frequency analysis have revealed that the number of significantly mutated genes (SMGs), which are somatically mutated at significantly higher rates than the background mutation rate in ECs, is the greatest in 21 cancer types [7]. Recently, several SMGs strongly associated with clinical cancer outcomes have been extensively characterized. For example, mutations in *FGFR2* may constitute a therapeutic target for ECs [8, 9]. *PIK3CA* mutations display less aggressive clinical behaviour [10]. Loss of *PTEN* expression may be associated with better overall survival in patients with the recurrence and metastasis of ECs [11–13]. Although previous studies have achieved great advances, a number of limitations still remain to be resolved. Due to that most of mutated genes in cancers are passenger genes that don’t promote tumorigensis, an effective method for identifying cancer-related genes among the large number of mutant genes is still needed. Furthermore, researchers are usually interested in SMGs associated with ECs and ignore low frequency or non-significantly mutated genes (non-SMGs) reported by the mutation annotation pipeline that could also be ECs-related genes. Among the mutated genes obtained from the annotated somatic mutation data (Level 2) on the TCGA data portal (http://cancergenome.nih.gov), the genes, which were not reported as SMGs, were defined as non-SMGs in our study. Therefore, elucidating the role of non-SMGs implicated with ECs tumorigensis, and discovering effective cancer diagnostic and therapeutic targets are crucial to improving the clinical outcome of ECs.

Next-generation sequencing (NGS) technology provides an important tool for cancer genome and genetic researches, uncovering a wide range of genetic aberrations that contribute to cancer development and progression. Recent studies utilizing the popular method of integrated RNA and DNA sequencing to identify cancer-related genes, have uncovered various gene mutations and expression mechanisms underlying tumorigenesis, progression, and prognosis [14–16]. Histological grade, histological type, and the International Federation of Gynecology and Obstetrics (FIGO) stage are important prognostic parameters for women with endometrial carcinoma [17–19]. Several studies have demonstrated the prognostic importance of histological grade, histological type, and FIGO stage [20, 21]. Depending on the three above pathological endpoints, the prognosis of EC patients varies significantly. Therefore, identifying biomarkers of potential use in targeted therapies and diagnosis of ECs is essential for the three pathological endpoints. Furthermore, recent research has shown that the variable combination population analysis (VCPA) algorithm [22], which considers the effects of variable combination, is an effective variable selection method. We used VCPA to discover the cancer-related non-SMGs from a large number of mutant genes.

Here, we proposed a strategy which integrates somatic mutations, RNA sequencing (RNA-Seq) gene expression data, and clinical data in The Cancer Genome Atlas (TCGA) Uterine Corpus Endometrial Carcinoma (UCEC) patients to identify cancer-related non-SMGs. In our study, we firstly found the non-SMGs by the mutation annotation analysis and performed differential expression analysis of non-SMGs between the different groups of each clinical endpoints. Clinical endpoints refers to histological grade, histological type, and FIGO stage of ECs. Then, VCPA method was further performed to select non-SMG associated with clinical phenotypes of ECs. As a result, there were 23, 19 and 24 non-SMGs selected by VCPA approach as the prognostic predictors for the histological grade, the histological type, and the FIGO stage, respectively. Importantly, most of these non-SMGs associated with clinical phenotypes of ECs have been reported in cancers or diseases. Our results indicated that non-SMGs may constitute potential cancer-related genes. Predictive models demonstrated that the non-SMGs associated with each clinical endpoint had a greater ability to distinguish the clinical phenotype of ECs compared with SMGs and can therefore be used as the potential biomarkers for cancer diagnosis and prognosis. These findings highlighted that the strategy proposed in our study can efficiently identify the important non-SMGs in cancers, which not only participate in the process of cancer progression, but may also serve as potential diagnostic biomarkers.

## Methods

### Tumour samples

Clinical data, somatic mutation data (Level 2) and RNA-Seq gene expression data (Level 3) of ECs were downloaded from the TCGA data portal (http://cancergenome.nih.gov) [23]. RNA-Seq gene expression data and somatic mutation data were generated using the Illumina Genome Analyzer platform.

### Mutation annotation

In order to identify mutations that may promote the initiation and progression of cancer, we used two popular prediction systems, namely Sorting Intolerant From Tolerant (SIFT) [24] and Polymorphism Phenotyping v2 (PolyPhen2) [25], both of which are available in the Annotate Variation (ANNOVAR) [26] website. In the SIFT program, a lower score indicates a greater probability of a deleterious mutation, while in PolyPhen2 a higher score indicates a greater probability of a deleterious mutation. We specified a non-synonymous single nucleotide variant (SNV) as deleterious if it had a SIFT score ≤ 0.05 or a PolyPhen2 score ≥ 0.5. Indels in the coding regions were all considered as deleterious. Similar to the previous study [27], our individual-based ‘deleterious mutation’ profile included deleterious missense SNVs, all other non-silent SNVs (nonsense, nonstop, splicing sites, and translation start sites), and all indels.

To further refine the deleterious mutation profile, the Catalogue of Somatic Mutations in Cancer (COSMIC) database [28], including mutations from EC tumour samples with matched normal samples, was subsequently used to identify mutations that were confirmed in ECs or reported in other cancers. In this study, if a gene occurred in at least one deleterious mutation that was confirmed in ECs or reported in other cancers, we considered this gene to be a damaging gene.

### Identification of non-SMGs that are closely related to clinical endpoints

We used the RNA-Seq data of the ECs in the TCGA portal to construct expression matrices. In our study, the mutated genes excluding the 58 SMGs (Additional file 1) in ECs, which had been reported in previous study [7], were defined as non-significantly mutated genes (non-SMGs). To identify non-SMGs associated with clinical endpoints of ECs, we conducted differential expression analysis and VCPA based on histological grade, histological type and FIGO stage of ECs separately.

Firstly, according to the EC histological grade (cell differentiation) information, we assigned EC patients into the low grade group (grade I and grade II endometrial adenocarcinomas (EACs)) and the high grade group (grade III EACs, high grade serous endometrial adenocarcinomas, and high grade mixed serous and endometrioid carcinomas). We also classified the ECs patients into, early stage (stage I-II) and advanced stage (stage III-IV) based on the FIGO stage. In addition, the EC patients were divided into Type I (estrogen related) (early stage and low grade EACs) and Type II (the non-estrogen related) (advanced stage and high grade EACs, serous endometrioid carcinomas, and mixed serous and endometrioid adenocarcinomas) based on their histological types. Then, for each clinical endpoint, the student’s t-test with false discovery rate (FDR)-adjusted *p* value <0.05 and fold change ≥2 (FC ≥ 2) or fold change ≤ 0.5 (FC ≤ 0.5) were used as the filtering criteria to select differentially expressed genes (DEGs) from the non-SMGs in damaging genes set. The same approach was used to identify DEGs from SMGs in damaging genes set. Previous research showed that the variable combination population analysis (VCPA) algorithm [22] can efficiently consider the effects of the feature combinations on the prediction models. Therefore, we used it to further identify the non-SMGs that are highly related to the clinical endpoints of ECs and their best combinations in predictive models. The MATLAB source code of VCPA can be downloaded from the website: https://cn.mathworks.com/matlabcentral/profile/authors/5526470-yonghuan-yun.

### Binary classification models for clinical endpoints

Support vector machine (SVM) has been applied extensively in the classification of two groups and is widely used in clinical endpoint prediction [29–33]. In this study, binary classification was conducted using libsvm3.17 [34] and the performance of the predictive models were assessed by the averaged areas under the receiver operating characteristic curve (AUCs). For the prediction of the histological grade, the histological type and the FIGO stage, we constructed the predictive models with the non-SMGs selected by VCPA as features. To determine the predictive ability of features, two thirds of the positive and negative samples were randomly selected as the training set, respectively, and the remaining positive samples and negative samples were used to build the test set. The model was constructed by using the training set with 10-fold cross-validation and then validated by using the test set. This process had been repeated for 100 times. To validate the ability of the features to discriminate the clinical phenotypes of ECs, SMGs were also used as features to develop predictive model with the same procedure.

To test the effectiveness of the selection of the features, for each of the clinical endpoints, we randomly selected the same number of genes from the non-SMGs lists as features to construct the predictive models. The entire process had also been repeated for 100 times.

### KEGG pathway enrichment analysis

Gene set enrichment analysis was performed using the online tool the Database for Annotation, Visualization and Integrated Discovery (DAVID) v6.8 [35, 36] (https://david.ncifcrf.gov/). This tool provides biological pathways annotation and biological processes (e.g., gene ontology (GO) terms). The biological pathways with *p* < 0.05 (Fisher’s exact test) [37] were considered as the significantly enriched Kyoto encyclopedia of genes and genomes (KEGG) pathways in our study.

## Results

### An overview of identifying important non-SMGs in ECs

**Fig. 1:**
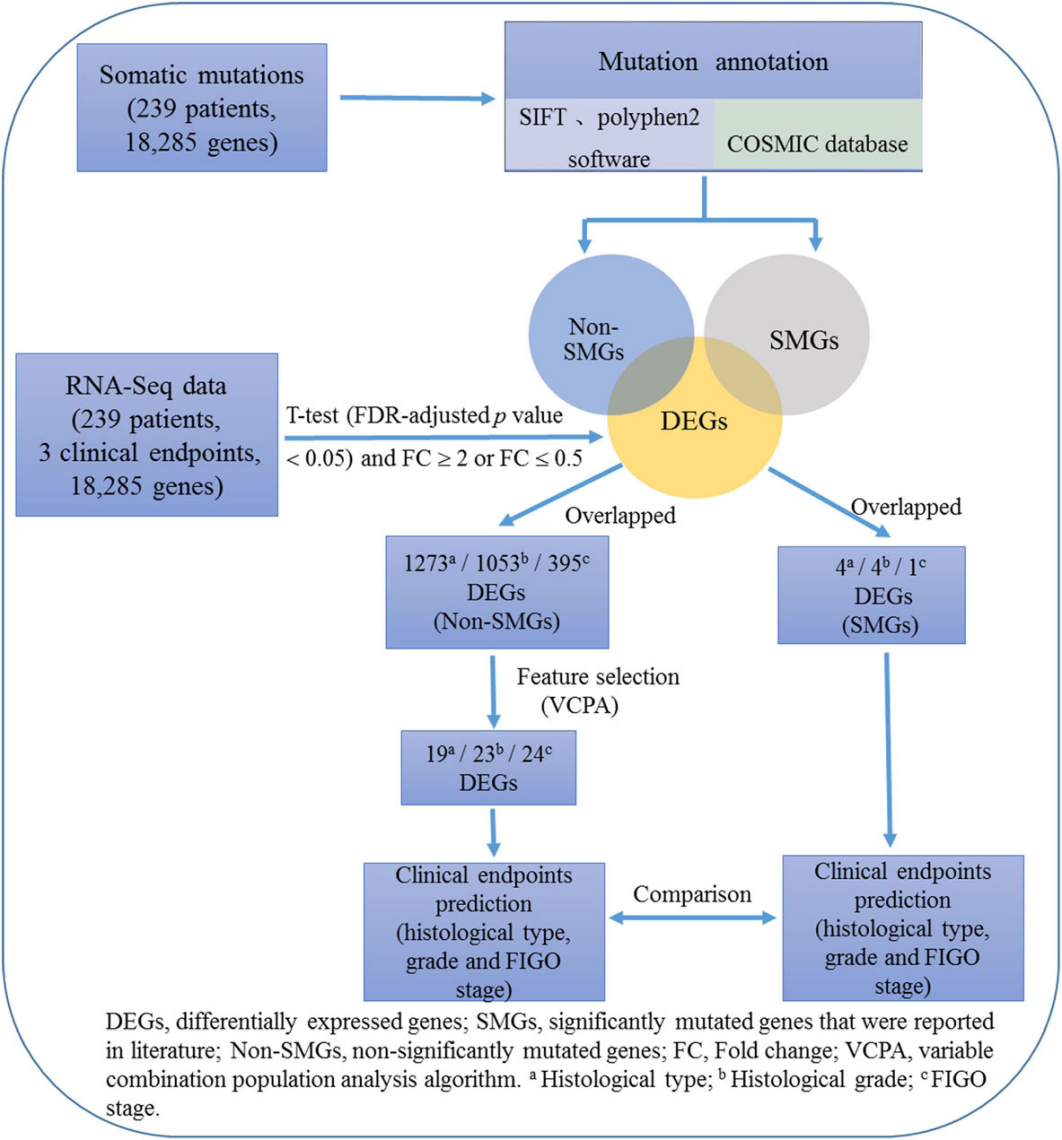
Framework for identifying the non-SMGs associated with clinical endpoints and validating their phenotypic relevance in ECs

In this study, we proposed a novel strategy to identify the important non-SMGs related to clinical endpoints of ECs (i.e., histological grade, histological type and FIGO stage) (Fig. 1). The strategy was mainly divided into four parts. For the 18,285 mutated genes with gene expression data, we firstly performed mutation annotation for gene mutations by SIFT, Polyphen2 and COSMIC database to found the damaging genes (including SMGs and non-SMGs). Secondly, differential expression analysis between the groups of patients with the same clinical endpoint was used to identify DEGs from 18,285 mutated genes. Then, for non-SMGs that were DEGs in damaging genes set, we used VCPA algorithm to further discover non-SMGs associated with each clinical endpoint of ECs, which were considered as the potential biomarkers in ECs. Finally, the potential biomarkers-based predictive models were constructed to discriminate the patients with different phenotypes in the clinical endpoint of ECs. 10-fold cross validation and AUCs were used to assess the performance of the models on training set and validation set, respectively. Moreover, to verify the biological function of potential biomarkers and the ability of them to distinguish the patients, we also used the features identified from the SMGs, which were reported by mutation annotation analysis, as features to develop the predictive models. Figure 1 presented a framework of the strategy in this study and the detailed description of each step was provided in the Methods.

### Identifying damaging genes

In our work, by integrating the somatic mutation profiles and RNA-Seq expression profiles of 239 EC samples, we detected 18,285 mutated genes with gene expression data in tumour samples. Following the annotations of their mutations, 9735 genes were identified as damaging genes. 54 out of 9735 damaging genes had been reported as SMGs in the previously study [7]. Therefore, 9681 genes were considered as non-SMGs.

### Identifying non-SMGs associated with histological grade of ECs

**Fig. 2:**
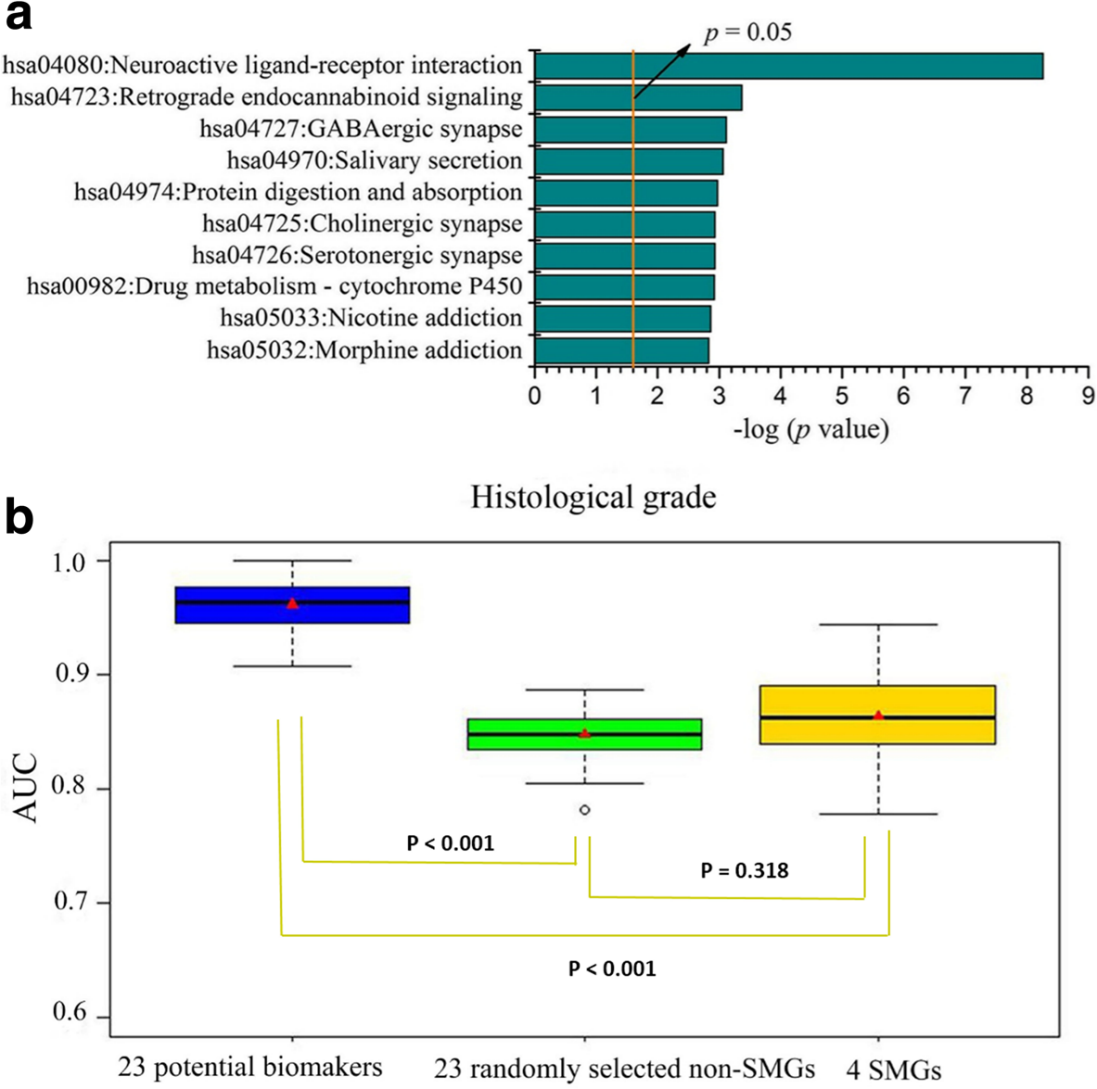
Significant KEGG pathways and the predictive model performance of non-SMGs associated with histological grade. **a** The KEGG pathways of the 1053 non-SMGs with the 10 lowest *p* values (*p* < 0.05). The *p* values were calculated using Fisher’s exact test and depicted on a log scale (−log_10_ *p* value). **b** The box plots of model performance on prediction the histological grade of ECs. Red triangles represent the average AUC. The *p* values were calculated based on a two-side Student’s t-test

For the 9681 non-SMGs that were found after annotation of gene mutations, we compared their expression levels between the low grade group and the high grade group. In total, 1053 non-SMGs were selected as DEGs (Additional file 2). Using the same method, 4 SMGs (*DNER, PIK3CA, SLC1A2, TPX2*) (Additional file 3) were also identified as DEGs from 54 SMGs. As shown in Fig. 2a, 1053 non-SMGs were significantly enriched in cancer-related or disease-related signaling pathways, including neuroactive ligand-receptor interaction signaling pathway (*p* < 0.001), calcium signaling pathway (*p* = 0.002), cAMP signaling pathway (*p* = 0.049), and retinol metabolism signaling pathway (*p* = 0.027). The top 10 significantly enriched KEGG pathways were shown in Fig. 2a and their detailed descriptions were listed in Additional file 4.

We performed VCPA to further select non-SMGs associated with histological grade from 1053 non-SMGs, and finally identified 23 non-SMGs that were considered as potential biomarkers (Additional file 5). Moreover, in order to determine whether the 23 potential biomarkers could be used as binary classification features and had better ability to distinguish the patients between low grade and high grade groups than the ability of SMGs, we examined 4 SMGs that were selected from 54 SMGs by differential expression analysis between low grade and high grade groups. The predictive models were constructed by using the 23 potential biomarkers and 4 SMGs as features, respectively. The prediction results of test set were shown in Fig. 2b. The predictive results of the 23 potential biomarkers were significantly superior to those of the 4 SMGs for the histological grade (two-sided t-test, *p* < 0.001, avg. AUC: 0.961 vs. 0.864).

To test the selection effectiveness of the 23 potential biomarkers, 23 genes were randomly selected from the 1053 non-SMGs as features and used to construct the predictive models. This process had been repeated for 100 times. The predictive results of the test set were shown in Fig. 2b. The 23 genes randomly selected from 1053 DEGs exhibited a weaker ability to predictive the histological grade (avg. AUC = 0.873) than the 23 potential biomarkers. These results indicated that the predictive ability of the 23 potential biomarkers was significantly superior to 23 genes that were randomly selected from 1053 non-SMGs (two-sided t-test, *p* < 0.001).

### Identifying non-SMGs associated with histological type of ECs

**Fig. 3:**
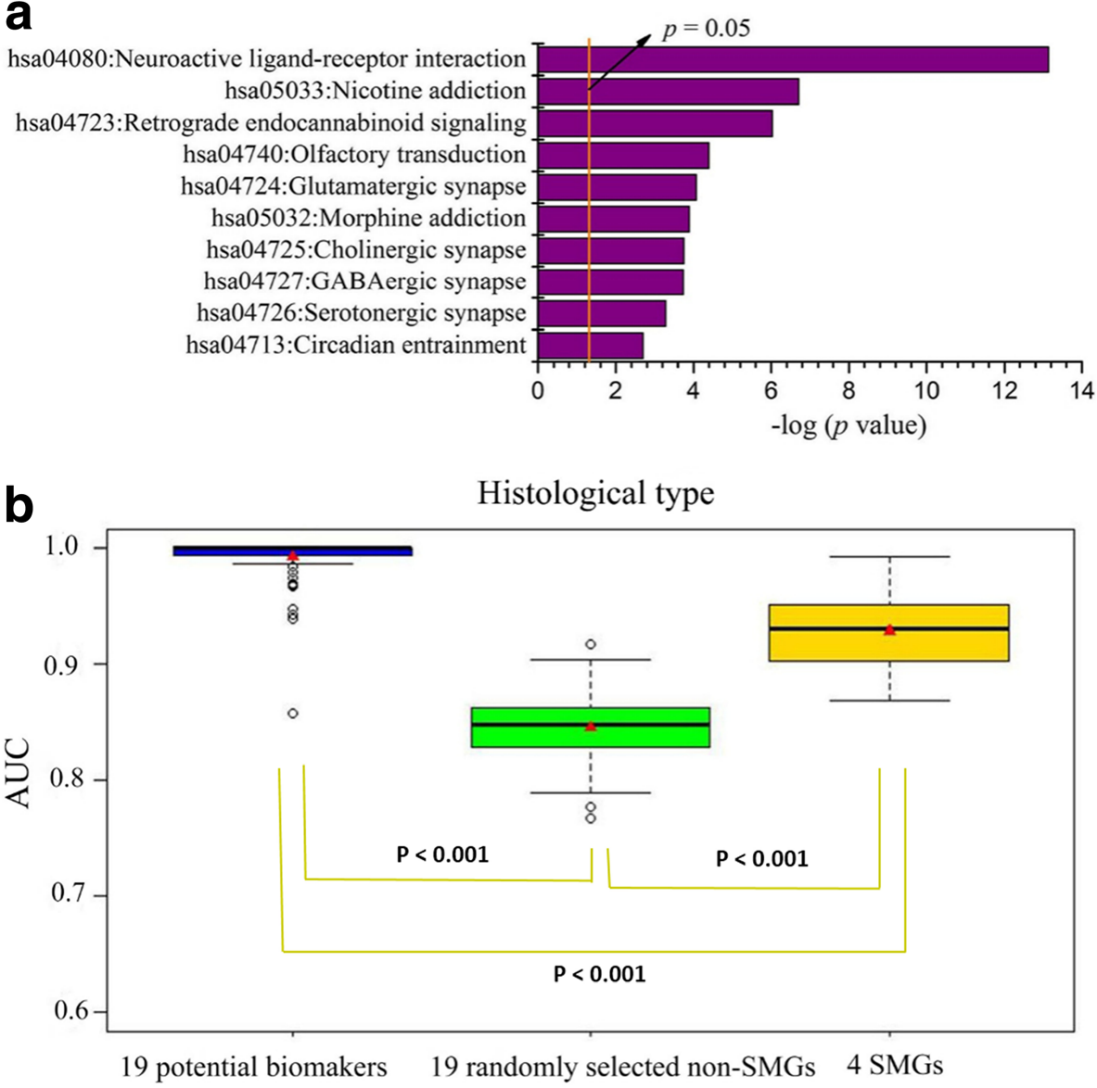
Significant KEGG pathways and the predictive model performance of non-SMGs associated with histological type. **a** The KEGG pathways of the 1273 non-SMGs with the 10 lowest *p* values (*p* < 0.05). The *p* values were calculated using Fisher’s exact test and depicted on a log scale (−log_10_ *p* value)). **b** The box plots of model performance on prediction the histological grade of ECs. Red triangles represent the average AUC. The *p* values were calculated based on a two-side Student’s t-test

The results of differential expression analysis between the Type I and Type II groups of ECs showed that 1273 out of 9681 non-SMGs (Additional file 6) and 4 out of 54 SMGs (Additional file 7) were significantly differentially expressed between the two patient groups. Gene set enrichment analysis revealed that 1273 non-SMGs were mainly involved in cancer-related or disease-related signaling pathways, including neuroactive ligand-receptor interaction signaling pathway (*p* < 0.001), calcium signaling pathway (*p* = 0.006), cAMP signaling pathway (*p* = 0.005), and retinol metabolism signaling pathway (*p* = 0.013). The 10 lowest *p* value KEGG pathways were shown in Fig. 3a. The KEGG pathways were detailed in Additional file 8.

Furthermore, 19 of 1273 non-SMGs were further identified by performing VCPA and were considered as potential biomarkers for histological type (Additional file 9). A predictive model with 19 non-SMGs as features was developed for predicting the histological type. To validate the ability of 19 non-SMGs to distinguish histological type, we also examined 4 SMGs (*DNER, TPX2, MYCN,* and *PIK3R1*) that were selected from 54 SMGs by differential expression analysis between Type I group and Type II group. The prediction results of test set for histological type were shown in Fig. 3b. It clearly showed that the model performance of 19 non-SMGs was significantly superior to the results of 4 SMGs (two-sided t-test, *p* < 0.001, avg. AUC: 0.993 vs. 0.928).

To verify the effectiveness of the proposed feature selection method, 19 genes were randomly selected from the 1273 non-SMGs and used as features to construct the predictive models. This procedure had been repeated for 100 times. Our results showed that the average AUC value for histological type of 19 potential biomarkers was significantly superior to the 19 non-SMGs that were randomly selected from the 1273 non-SMGs (two-sided t-test, *p* < 0.001, avg. avg. AUC: 0.993 vs. 0.866) (Fig. 3b).

### Identifying non-SMGs associated with FIGO stage of ECs

**Fig. 4:**
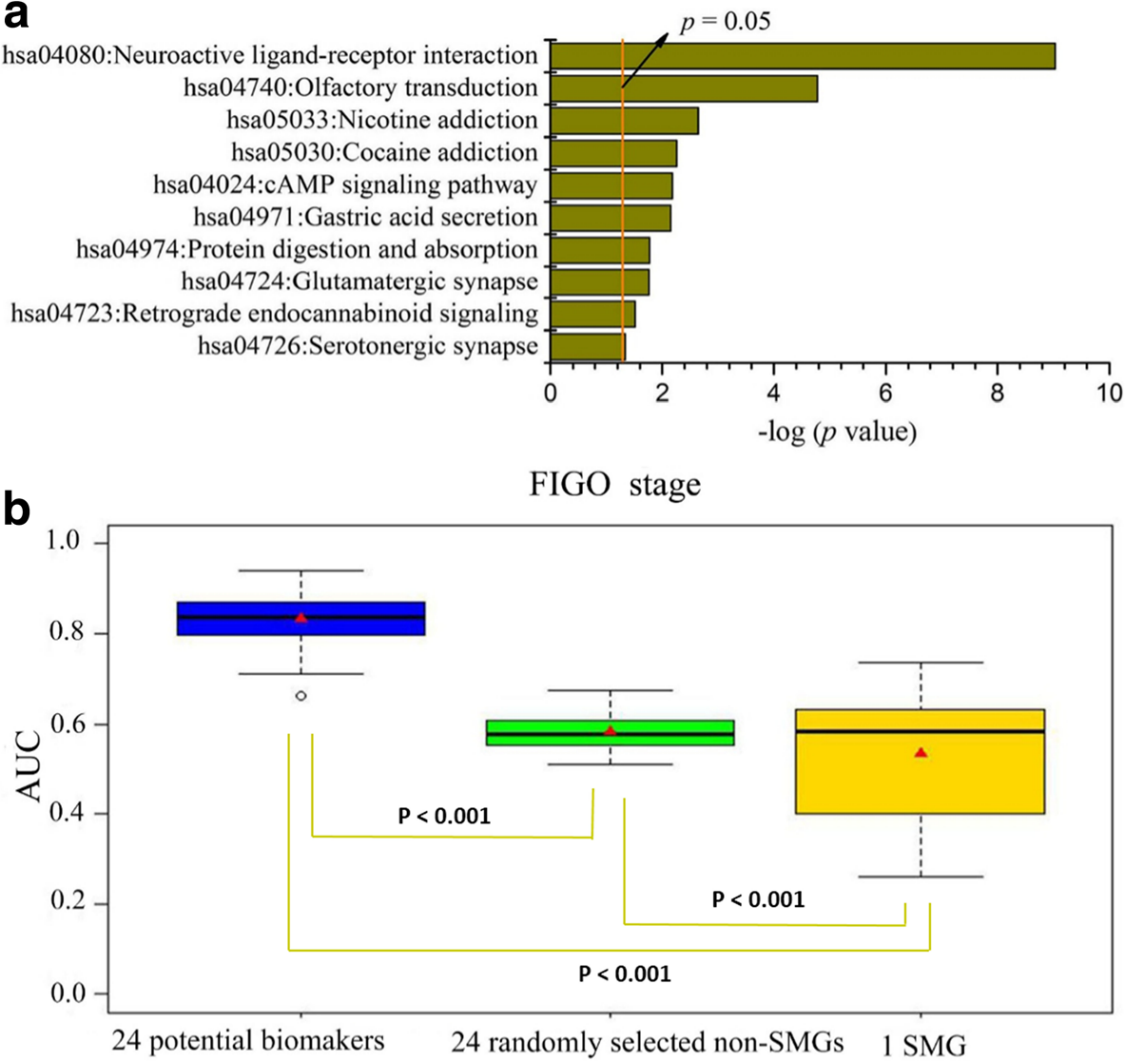
Significant KEGG pathways and the predictive model performance of non-SMGs associated with FIGO stage. **a** The KEGG pathways of the 395 non-SMGs (*p* < 0.05). The *p* values were calculated using Fisher’s exact test and depicted on a log scale (−log_10_ *p* value). **b** The box plots of model performance on prediction the histological grade of ECs. Red triangles represent the average AUC. The *p* values were calculated based on a two-side Student’s t-test

In the differential expression analysis between the early stage group and advanced stage group of ECs, we identified 395 non-SMGs (Additional file 10) from the 9681 non-SMGs, and 1 SMG (*DNER*) from the 54 SMGs. As shown in Fig. 4a, 395 non-SMGs were significantly enriched in neuroactive ligand-receptor interaction signaling pathway (*p* < 0.001) and cAMP signaling pathway (*p* = 0.007) (Fig. 4a). We found 24 non-SMGs (Additional file 11) that were considered as potential biomarkers by using VCPA, and then used them as features to build predictive model for predicting the FIGO stage. The prediction results were shown in Fig. 4b. The phenotypic (FIGO stage) relevance of 24 non-SMGs was significantly superior to 1 SMGs (*DNER*) (two-sided t-test, *p* < 0.001, avg. AUC: 0.832 vs. 0.535).

Moreover, we randomly selected 24 non-SMGs from the 395 non-DEGs as features to build predictive model with same method as that for the 24 potential biomarkers. The procedure had also been repeated for 100 times and the results are shown in Fig. 4b. As shown, 24 potential biomarkers had a significantly better ability to distinguish FIGO stage than random selection of 24 non-SMGs from the 395 non-DEGs (two-sided t-test, *p* < 0.001, avg. AUC: 0.832 vs. 0.606).

## Discussion

In this study, we examined the role of non-SMGs that were significantly differentially expressed between the patient groups in each clinical endpoint of ECs by combining the somatic mutations and gene expression analysis. Mutations, which make gene function loss and disrupt important biological processes, have a close relationship with tumorigenesis. Analysing gene expression levels can help us understand the mutation mechanism and identify cancer-related genes. Mutated genes cooperatively participate in the development and progression of cancer and may be highly correlated with the dysregulation of gene expression.

Patients with high grade and low grade EC exist clinical, morphological, and pathogenesis differences. Low grade patients are associated with favourable prognosis of ECs, while the prognosis in high grade group is generally poor [38–40]. It is crucial for ECs to select the appropriate diagnose and treatment option. In our study, 23 non-SMGs associated with histological grade were identified (Additional file 5) by VCPA and 16 out of them had been reported to be associated with various cancers or diseases. Among these genes, the gene *PSAT1* had been well studied in several cancers, such as the breast cancer, the lung cancer and the esophageal squamous cell carcinoma [41–43]. In breast cancer, over-expression of *PSAT1* was significantly associated with the malignant phenotype and survivals [41]. In lung cancer, *PSAT1* can promote cell invasion by activating MMP1 pathway and was found as a novel predictor in stage I non-small cell lung cancer [42]. In esophageal squamous cell carcinoma, *PSAT1* was identified as an potential anticancer therapeutic target [43]. Furthermore, *PAST1* can act as a subtype-specific biomarker that contributes to defining tumor histology at the molecular level [44]. The gene *TFAP2B*, for which the genetic variation was implicated with adipocytokine regulation and type 2 diabetes mellitus [45, 46], had been suggested to play a potential oncogenic role by regulating cancer cell growth and was previously identified as a promising therapeutic target for lung cancer [47]. Recent reports have displayed that *DCLK1* is a marker of differentiated cells and an epigenetic biomarker of intestinal cancer stem cell in colorectal cancer [48]. After annotation of gene mutations, 9 out of 12 EC patients harbouring *DCLK1* deleterious mutations were in the low grade group (Additional file 12). The expression of *DCLK1* was found to be up-regulated (T-test with FDR-adjusted *p* value <0.05, and FC > 2) in high grade EC patients in our study. These results suggested that *DCLK1* may be involved in cell differentiation of ECs and the expression of it was associated with high grade ECs. *NDST4* was previously identified as a putative tumor-suppressor gene in human colorectal cancer and its genetic loss might be related to the colorectal cancer progression [49]. In this study, we found that there were 4 low grade samples harbouring the deleterious mutations of *NDST4*, and the expression of *NDST4* was significantly up-regulated (T-test with FDR-adjusted *p* value <0.05, and FC > 2) in high grade EC patients. Therefore, the mutation of *NDST4* may be an important factor in EC development.

Histological type is an important predictor of the biological behavior of ECs, and our study identified 19 non-SMGs associated with histological type (Additional file 9). 15 out of 19 non-SMGs had been reported in previous studies as cancer-related or disease-related genes. The gene *BUB1*, which is one of the mitotic checkpoint genes, was associated with the histological differentiation, clinical stage and reduced postoperative survival of EC patients [50]. The high expression of *BUB1* was observed in gastric carcinomas [51], breast cancer [52] and have been reported to be involved in cancer cell differentiation [53]. Estrogen receptor 1 (*ESR1*) gene was a prognostic markers in ECs and had been suggested to play an important role in the progression of ECs [54]. Moreover, the gene expression levels of *ESR1* and *ESR2* had been found to be associated with the phenotype and survival of EC patients [55]. High expression levels of *ERS1* and *ERS2* were correlated with good prognosis of ECs. In our study, *ESR1* was significantly down-regulated (T-test with FDR-adjusted *p* value <0.05, and FC < 0.5) in Type II (the non-estrogen related, non-endometrioid) ECs. We then investigated whether 19 non-SMGs mutations had significantly difference on histological type. The sample distribution for the 19 non-SMGs with deleterious mutations was shown in Additional file 13. Our results demonstrated that *KCND3* and *ZNE804B* deleterious mutations significantly tended to occur in Type II EC patients (Fisher exact test, *p* = 0.004, *p* = 0.004, respectively), indicating the mutations of *KCND3* and *ZNE804B* may be involved in the progress of ECs.

Cancer stage is the most important indicator for diagnosis and adjuvant therapy of ECs. In this study, 24 non-SMGs associated with FIGO stage were selected (Additional file 11) and the sample distribution for the 24 non-SMGs with deleterious mutations was shown in Additional file 14. It was found that 16 out of 24 non-SMGs were associated with cancers or diseases. The gene *LHCGR* was associated with tumor metastasis that involved in cell growth and neoangiogenesis, and plays an important role in luteinizing hormone (LH) receptors, which may impact on the tumorigenesis of ECs. The expression of *LHCGR* was also correlated with cell proliferation of ECs [56, 57]. The up-regulated expression of *LHCGR* had been found in the malignant tissue comparing with the normal tissue [58]. The down-regulated expression of *RERGL* was related to poor prognosis in papillary thyroid cancer patients [59], and also implicated with advanced stage EC patients in our study. Yang et al. considered the gene *RERGL* as a potential tumor suppressor gene [60] because it shared some conserved regions with *RERG* [61]. Moreover, the deletion of *RERGL* had been reported in colorectal cancer. Backes et al. found that the gene *UQCRFS1* played an important role in promoting cell growth, and the genetic amplification or over-expression of it has been observed in multiple types of cancers, including breast cancer [62], ovarian cancer [63], gastric cancers [64]. In our study, the up-regulated (T-test with FDR-adjusted *p* value <0.05, and FC > 2) of *UQCRFS1* expression was significantly associated with the advanced stage ECs, suggesting it may contribute to the development of ECs.

In addition, the model performance on predicting the clinical endpoints by using SMGs as features was inferior to using the non-SMGs identified in our study, indicating that non-SMGs can be used as a good complement for cancer diagnosis and treatment. Further studies are still needed to better understand the biological functions of them, which can be helpful to identify the novel therapeutic targets for cancer prevention, diagnose and treatment. Note that, when using the SMGs as features, the insufficient model performance on predicting the clinical endpoints may be caused by the less number of SMGs in the models instead of indicating the irrelevant relationship between the SMGs and the ECs.

## Conclusions

In conclusion, similar to SMGs, non-SMGs also play an important role in ECs. By integrating somatic mutations and RNA-Seq data, we can effectively identify important non-SMGs in ECs which are closely related to the phenotypic characteristics in clinics and can be served as potential biomarkers for the prediction of FIGO stage, histological grade, and histological type of ECs.

## Additional files

Additional file 1: The list of SMGs in ECs that were collected from the website. (XLSX 10 kb)

Additional file 2: Summary of 1053 non-SMGs showed different expression patterns in the groups of histological grade patients. (XLSX 80 kb)

Additional file 3: Summary of 4 SMGs showed different expression patterns in the groups of histological grade patients. (XLSX 8 kb)

Additional file 4: The KEGG pathways with significant enrichment of 1053 non-SMGs. (XLSX 12 kb)

Additional file 5: Summary of the 23 non-SMGs that were identified from 1053 non-SMGs by using VCPA. (XLSX 11 kb)

Additional file 6: Summary of 1273 non-SMGs showed different expression patterns in the groups of histological type patients. (XLSX 95 kb)

Additional file 7: Summary of 4 SMGs showed different expression patterns in the groups of histological type patients. (XLSX 8 kb)

Additional file 8: The KEGG pathways with significant enrichment of 1273 non-SMGs. (XLSX 13 kb)

Additional file 9: Summary of the 19 non-SMGs that were found from 1273 non-SMGs by using VCPA. (XLSX 10 kb)

Additional file 10: Summary of 395 non-SMGs showed different expression patterns in the groups of FIGO stage patients. (XLSX 34 kb)

Additional file 11: Summary of the 24 non-SMGs that were found from 395 non-SMGs by using VCPA. (XLSX 11 kb)

Additional file 12: The distribution of samples harbouring the deleterious mutations of 23 non-SMGs in histological grade samples. (JPEG 76 kb)

Additional file 13: The distribution of samples harbouring deleterious mutations of 19 non-SMGs in histological type samples. (JPEG 79 kb)

Additional file 14: The distribution of samples harbouring deleterious mutations of 24 non-SMGs in FIGO stage groups. (JPEG 72 kb)

## Abbreviations

ANNOVAR: Annotate Variation
AUC: The Area under the receiver operating characteristic curve
COSMIC: Catalogue of Somatic Mutations in Cancer
DAVID: The Database for Annotation, Visualization and Integrated Discovery
DEGs: Differentially expressed genes
EACs: Endometrial adenocarcinomas
ECs: Endometrial cancers
ESR1: Estrogen receptor 1
FC: Fold change
FDR: False discovery rate
FIGO: International Federation of Gynecology and Obstetrics
GO: Gene ontology.
KEGG: Kyoto encyclopedia of genes and genomes
LH: Luteinizing hormone.
NGS: Next-generation sequencing.
Non-SMG: Non-significantly mutated gene.
Polyphen2: Polymorphism Phenotyping v2.
RNA-Seq: RNA sequencing.
SIFT: Sorting Intolerant from Tolerant.
SMGs: Significantly mutated genes.
SNV: Single nucleotide variant.
SVM: Support vector machine.
TCGA: the Cancer Genome Atlas.
UCEC: Uterine Corpus Endometrial Carcinoma.
VCPA: Variable combination population analysis
