# Supplementary figures and images for: Mutation status coupled with RNA-sequencing data can efficiently identify important non-significantly mutated genes serving as diagnostic biomarkers of endometrial cancer
Source: BMC Bioinformatics. 2017 Dec 28;18(Suppl 14):472. doi: 10.1186/s12859-017-1891-6 (PMC5751793; doi:10.1186/s12859-017-1891-6)

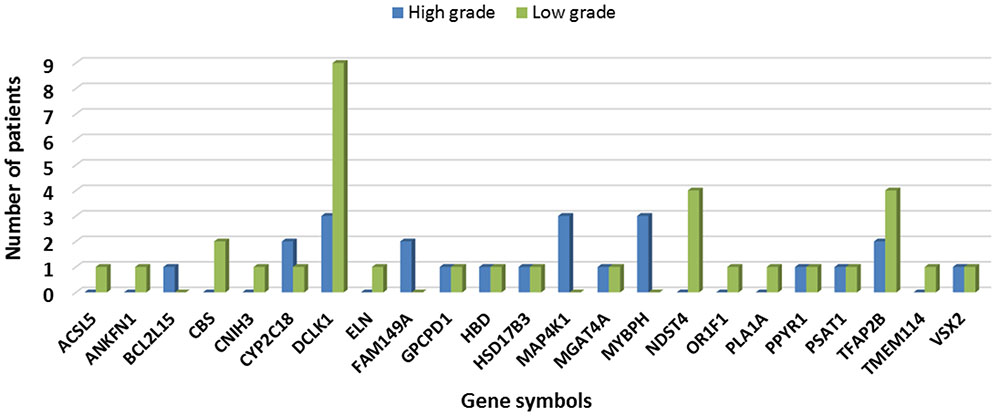

Supplement: Supplementary file 12 — The distribution of samples harbouring the deleterious mutations of 23 non-SMGs in histological grade samples. (JPEG 76 kb) [file 12859_2017_1891_MOESM12_ESM.jpg]

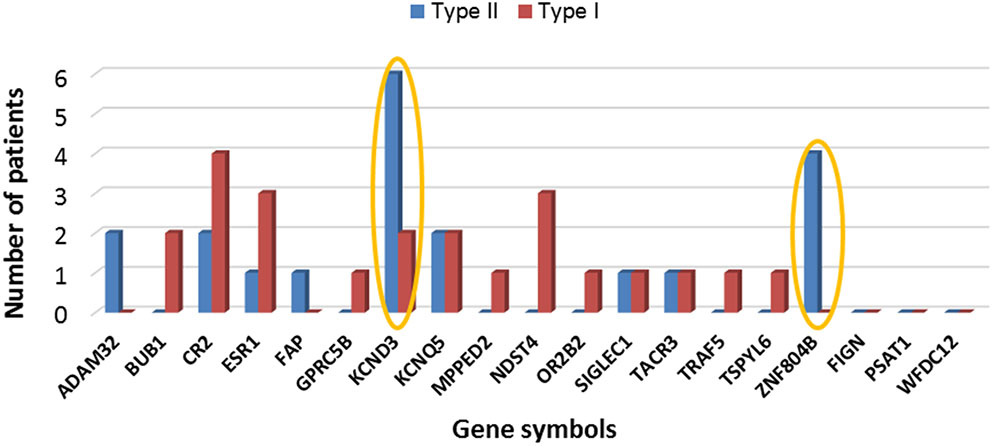

Supplement: Supplementary file 13 — The distribution of samples harbouring deleterious mutations of 19 non-SMGs in histological type samples. (JPEG 79 kb) [file 12859_2017_1891_MOESM13_ESM.jpg]

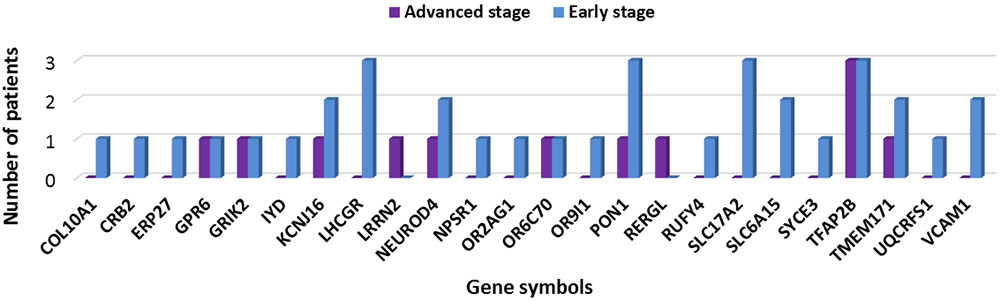

Supplement: Supplementary file 14 — The distribution of samples harbouring deleterious mutations of 24 non-SMGs in FIGO stage groups. (JPEG 72 kb) [file 12859_2017_1891_MOESM14_ESM.jpg]
